# Directed differentiation of human iPSC into insulin producing cells is improved by induced expression of PDX1 and NKX6.1 factors in IPC progenitors

**DOI:** 10.1186/s12967-016-1097-0

**Published:** 2016-12-20

**Authors:** Maciej P. Walczak, Anna M. Drozd, Ewelina Stoczynska-Fidelus, Piotr Rieske, Dawid P. Grzela

**Affiliations:** 1 Department of Research and Development, Celther Polska Ltd., Milionowa 23, 93-193 Łódź, Poland; 2 Department of Tumor Biology, Medical University of Łódź, Żeligowskiego 7/9, 90-752 Łódź, Poland; 3 Research and Development Unit, Personather Ltd., Milionowa 23, 93-193 Łódź, Poland

**Keywords:** Defined culture conditions, Diabetes, Differentiation, Induced pluripotent stem cells, Insulin producing cells, NKX6.1, PDX1, Reprogramming

## Abstract

**Background:**

Induced pluripotent stem cells (iPSC) possess an enormous potential as both, scientific and therapeutic tools. Their application in the regenerative medicine provides new treatment opportunities for numerous diseases, including type 1 diabetes. In this work we aimed to derive insulin producing cells (IPC) from iPS cells established in defined conditions.

**Methods:**

We optimized iPSC generation protocol and created pluripotent cell lines with stably integrated *PDX1* and *NKX6.1* transgenes under the transcriptional control of doxycycline-inducible promoter. These cells were differentiated using small chemical molecules and recombinant Activin A in the sequential process through the definitive endoderm, pancreatic progenitor cells and insulin producing cells. Efficiency of the procedure was assessed by quantitative gene expression measurements, immunocytochemical stainings and functional assays for insulin secretion.

**Results:**

Generated cells displayed molecular markers characteristic for respective steps of the differentiation. The obtained IPC secreted insulin and produced C-peptide with significantly higher hormone release level in case of the combined expression of *PDX1* and *NKX6.1* induced at the last stage of the differentiation.

**Conclusions:**

Efficiency of differentiation of iPSC to IPC can be increased by concurrent expression of *PDX1* and *NKX6.1* during progenitor cells maturation. Protocols established in our study allow for iPSC generation and derivation of IPC in chemically defined conditions free from animal-derived components, which is of the utmost importance in the light of their prospective applications in the field of regenerative medicine.

**Electronic supplementary material:**

The online version of this article (doi:10.1186/s12967-016-1097-0) contains supplementary material, which is available to authorized users.

## Background

Type 1 diabetes (T1D) is one of the most frequent chronic autoimmune diseases diagnosed among juveniles, and its global incidence continues to rise [1]. This condition is characterized by pancreatic beta cell damage leading to insufficient insulin production and altered carbohydrate metabolism. It develops at an early age and requires constant treatment, which generates substantial costs and lowers quality of life. Commonly, therapy of type 1 diabetes is based on supplementation of deficient hormone in form of regular injections. However, this method does not address the cause of the disease, regarded as a lack of functional intrinsic mechanisms providing carbohydrate homeostasis.

Pancreatic islet transplantation is another approach to T1D treatment. Its application is however limited due to complex medical procedure and shortage in the number of islets donors. Moreover, it is not a permanent solution since patients who are forced to undergo several procedures eventually need to receive insulin injections due to the destruction of the graft by the immune system [2].

A concept of a therapy based on differentiated induced pluripotent stem cells (iPSC) raises great promises in the field of T1D treatment. These cells are generated from somatic cells by forced expression of transcription factors characteristic for embryonic stem cells [3]. Numerous investigations have been conducted to confirm resemblance between iPSC and embryonic stem cells (ESC) isolated from the inner cell mass of developing embryo [4, 5].

These cells are characterized by features of great importance in terms of disease treatment, such as the fact that they are obtained from patient’s own cells. This trait eliminates risk of potential stem cell-based graft rejection [6].

A number of research teams are focused on generation of functional insulin producing cells (IPC) that could replace damaged beta cells. Their approaches are based on mimicking of in vivo mechanisms underlying pancreas development [7, 8]. However, at present there is no sufficient comprehension of the molecular backgrounds of pancreatic organogenesis, which originates from complexity of this process and difficulties in exploring it in humans. Therefore, despite of numerous attempts, an efficient protocol of functional insulin producing cells for clinical applications has not been established [9].

The main obstacle is caused by heterogeneity of obtained cells as well as their functionality, which typically results in production of other pancreatic hormones or inadequate response to changing glucose concentration in vitro [10–12]. Thus, the on-going research on generation of clinically applicable insulin producing cells continues all around the world.

In this work an attempt has been made to generate insulin producing cells from induced pluripotent stem cells by means of chemical differentiation and induction of certain transgene expression [13]. For the purpose of the study, selected genes are *PDX1* (Pancreatic and Duodenal Homeobox 1) and *NKX6.1* (Homeobox Protein Nkx6.1), as their expression seems to be critical in pancreatic organogenesis, which was reported in numerous studies [14, 15]. Moreover, these factors act on different stages of pancreatic development, and as indicated in several investigations, disturbances of their expression result in altered organ structure or functional activity of the pancreas [16].

**Fig. 1:**
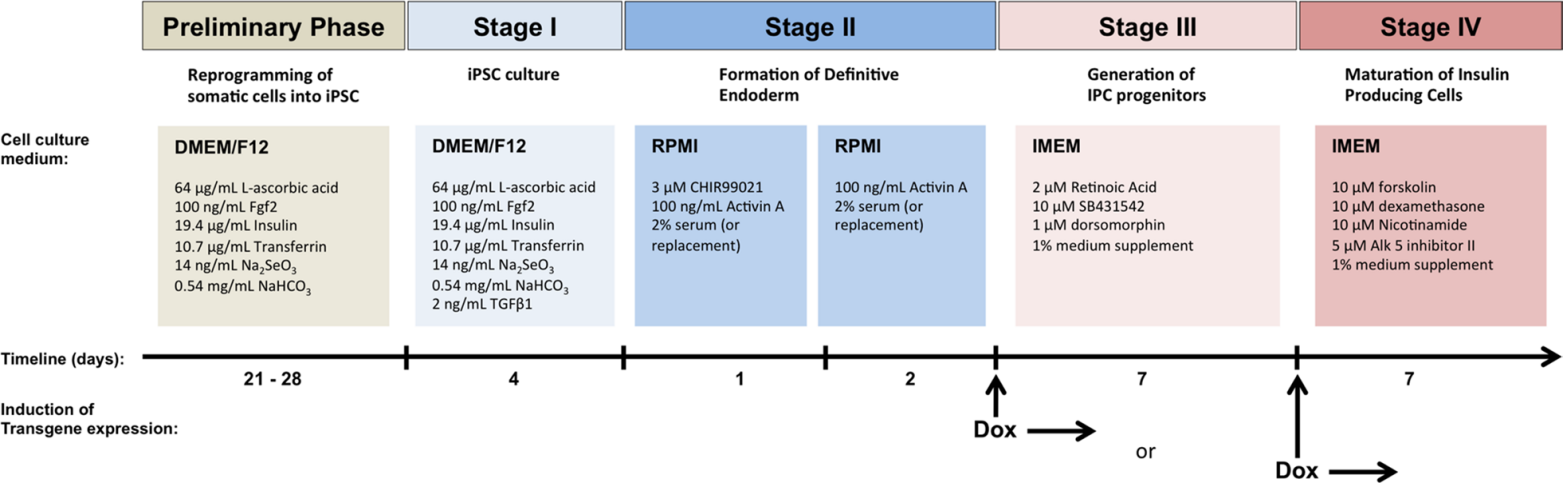
The outline of the differentiation procedure of iPSC into insulin producing cells. Schematic representation of the procedure used for differentiation of iPS cells to insulin producing cells. *Arrows* indicate time points of induction of transcription factors expression with doxycycline (DOX). The experimental framework consisted of four stages: culture of iPS cells, generation of definitive endoderm cells, formation of pancreatic progenitor cells and development of mature insulin producing cells. The diagram presents composition of the media used at each step

Considering prospective clinical application, it is essential to obtain these cells in conditions that will ensure patient’s safety. Therefore, attempts have been made to establish protocols and defined conditions of iPSC generation, culture and differentiation. The framework of the experimental design is shown in Fig. 1.

In order to investigate the influence of over-expression of PDX1 and NKX6.1 factors on generation of insulin producing cells in vitro, genetically engineered iPS cell lines with introduced sequences of *PDX1*-*VP16, NKX6.1* and combination of thereof under control of inducible promoter were established. Moreover, the expression was induced on selected stages of differentiation process.

This work focuses on three aspects. Firstly, reprogramming of selected somatic cell lines to iPSC in defined conditions. Secondly, generation of definitive endoderm cells (DE) from iPSC by activation of TGFβ signalling pathway and inhibition of GSK3β in presence of either human or bovine serum or combination of defined factors. And finally, we analysed the influence of PDX1 and NKX6.1 transcription factors on the process of differentiation and maturation of insulin producing cells.

## Methods

### Reagents

Unless specified otherwise, all chemicals were from Sigma-Aldrich (St. Louis, MO, USA). Cell culture media were purchased from Life Technologies (Carlsbad, CA, USA). Restriction endonucleases, polymerases and DNA modifying enzymes were from New England Biolabs (Ipswitch, MA, USA).

### DNA constructs

To create pENTR/zeo-Pdx1-VP16 vector, Pdx1 coding region in frame with VP16 trans-acting coding sequence from the *Herpes Simplex* virus was synthesised by GeneArt AG (Regensburg, Germany) as a String™ DNA, and amplified with Q5 DNA polymerase using Pdx1-VP16 attB1 and Pdx1-VP16 attB2 oligonucleotides. The PCR product was shuttled with Gateway BP Clonase II (Life Technologies) into pDONR/zeo plasmid (Life Technologies) resulting in pENTR/zeo-Pdx1-VP16 construct. The sequence was confirmed by DNA sequencing with M13 Fwd (−20) and M13 Rev primers. To generate pENTR/zeo-Nkx6.1 construct, the total RNA was isolated from HEK293T cells and reverse-transcribed with random hexamer primers and M-MuLV reverse transcriptase. Nkx6.1 coding sequence was amplified from the cDNA with Q5 DNA polymerase with Nkx6.1 attB1 and Nkx6.1 attB2 primers and transferred into pDONR/zeo plasmid by means of the Gateway BP Clonase II enzyme to create pENTR/zeo-Nkx6.1 vector. In order to generate pLVX-TRE3G-DEST construct, pLVX-TRE3G-IRES plasmid (Clontech, Mountain View, CA, USA) was digested with *Eco*RI and *Bam*HI restriction enzymes and filled with T4 DNA polymerase. The vector was isolated from the agarose gel with QIAquick DNA Gel Extraction Kit (Qiagen, Venlo, Limburg, The Netherlands) and ligated using T4 DNA ligase with Gateway Rf.A cassette (Life Technologies) that encompasses attR1 and attR2 recombination sequences, chloramphenicol resistance gene and ccdB negative selection marker. The correct orientation of the ligated insert was determined by restriction digest and DNA sequencing using primers Gateway 1 and Gateway 2. The transfer of Pdx1-VP16 fragment from the ENTRY construct into the final pLVX-TRE3G-Pdx1-VP16 vector was done using Gateway LR Clonase II enzyme (Life Technologies). The pLVX-TRE3G-Nkx6.1 plasmid was created by shuttling of Nkx6.1 sequence from the pENTR/zeo-Nkx6.1 vector into pLVX-TRE3G-DEST with Gateway LR Clonase II. The identity of the last two vectors was determined by restriction digest and DNA sequencing using pLVX-TRE3G Fwd and pLVX-TRE3G Rev primers. To generate pCXB-EmGFP vector, EmGFP coding sequence was PCR-amplified from pRSET-EmGFP plasmid (Life Technologies) with FP attB1 and FP attB2 primers. Obtained PCR product was shuttled into pDONR/zeo plasmid with application of Gateway BP Clonase II enzyme, resulting in pENTR/zeo-EmGFP construct. The transfer of the EmGFP fragment from the ENTRY construct into final expression vector was done by with Gateway LR Clonase II enzyme and pCXB-DEST plasmid (described in [17]). DNA sequences of oligonucleotides used for cloning purposes are included in Additional file 1: Table S1.

### Generation and culture of induced pluripotent stem cells

Unless stated otherwise, iPS cells were generated as described previously [17]. Briefly, cells were seeded on Geltrex-coated cell culture plates and incubated overnight in a suitable primary medium. On the next day, cells were transfected with five episomal plasmids (pCE-hOct3/4, pCE-hSK pCE-hUL, pCE-mp53DD, and pCXB-EBNA1). After a 24-h incubation, medium was replaced with TeSR-E7 medium (StemCell Technologies), and the transfection was repeated. The medium was changed every other day for 14 days. After that, culture medium was replaced with Essential 8 and cells were cultivated until iPSC colonies started to appear. Then, they were transferred onto a new, Geltrex-coated culture dishes, expanded and maintained in Essential 8 medium.

### Cell culture

iPS cells derived from human fibroblasts, established as described earlier [17], were cultured in low oxygen conditions (5% O_2_/5% CO_2_), in chemically defined Essential 8 medium on cell culture plates coated with Geltrex (20 μg/cm^2^) or recombinant Vitronectin (0.5 μg/cm^2^). Cells were passaged twice a week when they reached 60–70% confluence. Pluripotent cells were incubated in Essential 8 medium supplemented with 10 μM Y-27632 for 1 h before passaging and colony expansion. Cells were dissociated with 0.5 mM EDTA in ion-free PBS for 5–8 min at room temperature. Afterwards, EDTA solution was removed, and the process of cell separation was terminated by addition of Essential 8 medium. Clumps of cells were then transferred into fresh culture vessels coated with Geltrex or Vitronectin. Renal epithelial cells were isolated from urine samples according to the protocol described previously [18]. Cells were cultured on gelatin-coated cell culture plates and maintained in REBM medium supplemented with REGM SingleQuot Kit (Lonza, Basel, Switzerland). HFF-1 (neonatal human foreskin fibroblasts) and HEK293T cell lines were obtained from ATCC (Manassas, VA, USA). Cells were cultured in DMEM supplemented with 10% FBS. HeLa cells were purchased from DSMZ (Braunschweig, Germany) and cultured in DMEM medium supplemented with 10% FBS. In addition, all the abovementioned cell culture media contained penicillin (100 U/mL), streptomycin (100 µg/mL), amphotericin B (0.25 µg/mL) and 2.5 µg/mL of Plasmocin (InvivoGen, San Diego, CA, USA).

### Coating agents

Geltrex™ hESC-qualified basement membrane matrix and Coating Matrix Kit (human, recombinant Colagen I) were purchased from Life Technologies. Vitronectin XF™, full-length human recombinant protein was obtained from STEMCELL Technologies (Vancouver, BC, Canada). Laminin-511 and Laminin-521 was purchased from BioLamina AB (Sundbyberg, Sweden). Fibronectin from human plasma, Laminin from Engelbreth-Holm-Swarm murine sarcoma basement membrane, Collagen IV from human placenta and porcine Gelatin were from Sigma-Aldrich. All of the protein coating agents were diluted in DMEM/F12 basal medium to desired concentrations, applied on the surfaces of cell culture vessels and incubated for 1 h at 37 °C.

### Preparation of L64-PEI copolymer

L64-PEI copolymer was synthesised as reported earlier [19, 20] with minor modifications. Briefly, 2 g of pluronic-L64 PEG-PPG-PEG block polymer, with average molecular weight of 2900 Da, was desiccated in a vacuum dryer (VacuCell 22, MMM Medcenter, Germany) at 40 °C overnight and activated with threefold molar excess of 1,1′-carbonyldiimidazole (CDI) in 10 mL of anhydrous acetonitrile. After 3 h of incubation on shaker platform at room temperature, the reaction mixture was diluted with equal volume of deionized water and dialyzed against 10% ethanol/H_2_O for 24 h using 1 kDa molecular weight cut-off cellulose membrane tube. Following dialysis, the activated Pluronic-L64 derivate was desiccated in a vacuum dryer overnight at 40 °C and dissolved in 20% ethanol/H_2_O. The activated polymer was conjugated with fivefold molar excess of polyethyleneimine (PEI, molecular weight 1200 Da) and the resulting solution was stirred overnight at room temperature. In order to remove unincorporated PEI, the reaction mixture was dialyzed for 48 h at room temperature against deionized water using 3.5 kDa cut-off cellulose membrane. To separate conjugated polymer from unreacted Pluronic-L64, the reaction product was processed with HiTrap SP cation exchange chromatography column (GE Healthcare Bio-Sciences, Uppsala, Sweden), washed with five column volumes of the 10% ethanol/H_2_O and eluted with 500 mM citric acid. Following 24 h of dialysis with 3.5 kDa MWCO cellulose membrane against 20% ethanol/H_2_O, the reaction product was desiccated for 24 h in vacuum dryer, heated at 65 °C in order to remove residual DNAse activity, weighted and dissolved in nuclease-free water.

### Production of lentiviral vectors

Lentiviral particles were generated by the simultaneous transient transfection of HEK293T cells with packaging plasmid (pLV-HELP), envelope plasmid (pLV-iVSV-G, for pseudotyping viral particles with pantropic VSV-G protein) and the transfer vector carrying the gene of interest. Packaging and envelope plasmids were purchased from InvivoGen as a part of LENTI-Smart kit, a 2nd generation lentiviral system. HEK293T cells were seeded at a density 8 × 10^6^ cells per 10 cm dish and grown in DMEM (high glucose, 4.5 g/L) supplemented with 10% FBS. 12–16 h after the initial plating, culture media were discarded and replaced with 12 mL of pre-warmed growth medium (DMEM with 10% FBS). HEK293T cells were transfected with 30 μg of plasmid DNA (15 μg of transfer vector, 10 μg of packaging plasmid and 5 μg of envelope-encoding plasmid) using linear polyethylenimine (PEI, 25 kDa, Polysciences, Warrington, PA, USA) at 2:1 PEI to DNA ratio. One day after transfection, culture media were replaced with fresh ones, and additionally supplemented with 10 mM HEPES buffer (pH = 7.2). Cell supernatants containing lentiviral particles were harvested after 24 and 48 h. Cellular debris was removed from the supernatants by centrifugation (2000G, 5 min) and filtration through a 0.45 μm PES (low protein binding) filter. Afterwards, lentiviral particles were concentrated by ultrafiltration with 100 kDa cut-off Amicon centrifugal filter (EMD Millipore, Billerica, MA, USA), aliquoted and stored at −80 °C.

### Lentiviral transduction of iPS cells

In order to create pluripotent lines expressing Tet-On transactivator gene, iPS cells were seeded in low density on Geltrex-coated (20 μg/cm^2^) 6-well plates and cultured in Essential 8 medium. After 24 h, the medium was changed and approximately 100 transduction units of lentiviral particles prepared from pLVX-EF1α-Tet3G transfer plasmid (Clontech) was added to the culture medium. After 2 days, the medium of transduced iPS cells was changed to Essential 8 medium supplemented with 50 μg/mL of G418 (Life Technologies). Cells were maintained in selection medium until non-transduced iPS cells in control well were no longer detected on the plate. G418 resistant iPS cell colonies carrying Tet-On 3G transactivator gene were picked and expanded around day 10 post transduction.

To establish iPSC lines carrying Pdx1 and Nkx6.1 transgene under the control of doxycycline-inducible promoter, iPSC line with stably integrated Tet-on 3G regulatory gene were seeded at low density on Geltex-coated 6-well plate. Next day, cells were transduced with lentiviral vectors carrying either Pdx1-VP16 or Nkx6.1, or the combination of both under the control of doxycycline-regulated TRE3GV promoter. 2 days after the transduction culture medium was supplemented with 20 ng/mL of Puromycin (InvivoGen), and iPS cell colonies were maintained in antibiotic-containing media until non-transduced cells were no longer observed in control wells. 7 days post transduction, puromycin-resistant iPSC colonies were isolated, expanded and examined for doxycycline-induced transgene expression.

### Alkaline phosphatase staining

Cells were fixed with 4% PFA in PBS for 20 min. Then, they were washed with TBS twice and stained with 5-bromo-4-chloro-3-indolylphosphate (BCIP) and nitro-blue tetrazolium (NBT) substrate solution (0.02% BCIP, 0.03% NBT, 5 mM MgCl_2_ in 150 mM TBS, pH 9.5) at 37 °C until the purple colour emerged, washed with distilled water, and dried.

### Flow cytometry

HeLa cells transfected with pCXB-EmGFP plasmid were analysed for green fluorescent protein expression by flow cytometry. 2 days after transfection, cells were detached from cell culture vessels to a single-cell suspension with TrypLE Select (Life Technologies) and analysed using Amnis FlowSight flow cytometer (EMD Millipore). The acquisition was set for 70,000 events per sample, and the data were analysed using IDEAS software (EMD Millipore).

### xCELLigence impedance analysis of cells growth and survival

The RTCA xCELLigence (Roche, Basel, Switzerland) was used to determine cell viability. 150 μL of prepared culture medium was added into each well of E-plate 16. The background impedance was measured for 60 s. Fibroblasts and renal epithelial cells were grown on tissue culture vessels prior to the experiment. After reaching 50% confluence, they were rinsed with PBS, and detached from plates by treating them with Accutase. Single cell suspension was prepared and the cells were counted on ADAM-MC Cell System (NanoEnTek, Seoul, Korea). The cell suspension was added to medium-containing wells on E-plate 16 to final density 10,000 cells per well. The adhesion and cell viability was monitored every 60 min for a period of up to 72 h. The electrical impedance was quantified by the RTCA system as Cell index (CI) values. As the electrical impedance depends on the number of cells attached to the electrode and the dimensional changes of the attached cells, CI values represented physiological state of analysed cells.

### Immunofluorescence analysis

For immunofluorescence studies, cells were fixed for 20 min at room temperature with 4% paraformaldehyde (PFA) in PBS, and washed once with TBS-T (Tris-buffered 0.9% saline with 0.05% of Tween-20, pH = 7.2). After fixation, cells were permeabilized (for 10 min with TBS-T/0.25% Triton X-100) and blocked with 10% donkey serum in TBS-T. Cells were incubated with primary antibody (1 h, room temperature), washed three times with TBS-T and incubated for 1 h at room temperature with Alexa-conjugated secondary antibodies (Life Technologies) and 4′,6-diamidino-2-phenylindole (DAPI) (50 ng/ml). Coverslips were mounted in 10% polyvinylalcohol in 25 mM Tris–HCl (pH 8.7) with 5% glycerol and 2.5% 1,4-diazobicyclo[2,2,2]-octane. Samples were examined on an Eclipse Ci-S epifluorescence microscope equipped with a DS-5Mc colour CCD camera (Nikon, Tokyo, Japan). Images in blue channel (350/50ex, 400lp, 460/50em), green channel (480/40ex, 510lp, 535/50em), and red channel (560/40ex, 585lp, 630/75em) were acquired with NIS-Elements 4.0 and processed with ImageJ software. Antibodies used for immunofluorescence analysis are listed in Additional file 2: Table S2.

### Dithizone stainings

Dithizone stock solution was prepared from 10 mg of dithizone dissolved in 10 mL of DMSO. Cells on the culture dish were fixed with 4% PFA for 15 min at room temperature, rinsed with TBS-T and stained with dithizone working solution (1:100 dilution of the stock in PBS) for 30 min at 37 °C. After that, cells were washed two times with PBS and examined under a microscope.

### Quantitative real time PCR

Total RNA was isolated with a NucleoSpinTriPrep kit (Macherey–Nagel, Düren, Germany) according to manufacturer’s protocol. Concentration of nucleic acid samples was measured spectrophotometrically (NanoPhotometer, Implen, Germany) and 250 ng of RNA was reverse-transcribed using random hexamer primers and M-MuLV reverse transcriptase according to manufacturer’s specifications (New England Biolabs). 50 ng of cDNA template along with 200 nM of primer and 6 μL of SYBR Select Master Mix (Life Technologies) was used in reaction. Primer sequences are listed in Additional file 3: Table S3. Quantitative real-time PCR was performed on StepOnePlus™ Real-Time PCR System (Applied Biosystems, Foster City, CA, USA). In order to confirm the specificity of amplification, the melting curve was analysed in each case using StepOne™ Software v2.2.2 (Applied Biosystems). Normalized relative expression level was calculated utilizing the method described previously by Pfaffl [21] based on sample’s average C_T_ value and PCR efficiency.

### Differentiation of iPSC into insulin producing cells

The protocol of iPSC differentiation into insulin producing cells was based on the modified procedure described by Kunisada et al. [13]. Definitive endoderm was obtained using the following method. iPS cells with genetically introduced transgenes were seeded onto a 6-well plate covered with Geltrex or Vitronectin. Cells were maintained in Essential 8 medium for the next 4 days (with daily medium exchange) until they reached 70–80% of confluence. Then, the culture medium was changed to RPMI-1640 supplemented with 100 ng/mL of Activin A, 3 μM of CHIR99021 and 2% foetal bovine serum. iPS cells were maintained in abovementioned medium for 24 h to initiate the differentiation towards endodermal lineage. Afterwards, cells were incubated in RPMI-1640 basal medium supplemented with 100 ng/mL of Activin A and 2% of FBS for the next 2 days.

Definitive endoderm obtained in such manner was further differentiated in iMEM medium supplemented with 10 μM SB431542, 1 μM dorsomorphin, 2 μM retinoic acid and 1% of B27 or defined NS21 supplements (R&D Systems, Minneapolis, MN, USA). The cells were cultured in this medium for 7 days with medium exchange every other day. After that, cells were incubated in iMEM containing 10 μM forskolin, 10 μM dexamethasone, 10 μM nicotinamide, 5 μM Alk 5 inhibitor II and 1% of medium supplement (B27 or NS21). In order to induce TET promoter-controlled transgene expression, 10 ng/mL of doxycycline was added do the IPC differentiation media either through stage III or through stages III and IV.

### ELISA assays

Cells grown in culture conditions inductive for pancreatic islets specification were preincubated in L15 medium without insulin and supplemented with 2.5 mM glucose in 37 °C for 4 h. Supernatants were collected, filtered through 0.45 µm filter and used for determination of insulin secretion. For ELISA experiments, wells of 96-well plate (Human Insulin ELISA kit, Sigma-Aldrich) coated with antibodies against human insulin were incubated with 100 µL of analysed supernatants along with insulin concentration standards run in parallels, for 2.5 h with gentle shaking. The whole assay was performed at room temperature. After the incubation, solutions were discarded and wells were rinsed three times with 1× Wash buffer. Afterwards, 100 µL of the detection buffer containing biotinylated antibodies was applied to each well and incubated for 1 h. After the washing step (three times with 1× Wash buffer), bound antibodies were detected with streptavidin-conjugated horse-radish peroxidase (100 µL of the solution with 45 min of incubation). The reaction was visualised by incubation with 100 µL of colorimetric TMB substrate (30 min, development was ended with 50 µL of Stop solution. Absorbance was determined spectrophotometrically at 450 nm on NanoPhotometer (Implen).

### Statistical analyses

The reprogramming assays, xCELLigence, qPCR and ELISA experiments were carried out at least three times and presented as the average values ± SEM (standard error of the mean). Difference between samples was compared by the two-tailed Student’s t test and was considered significant at p < 0.05.

## Results

### Reprogramming of somatic cells to pluripotent state in defined culture conditions

**Fig. 2:**
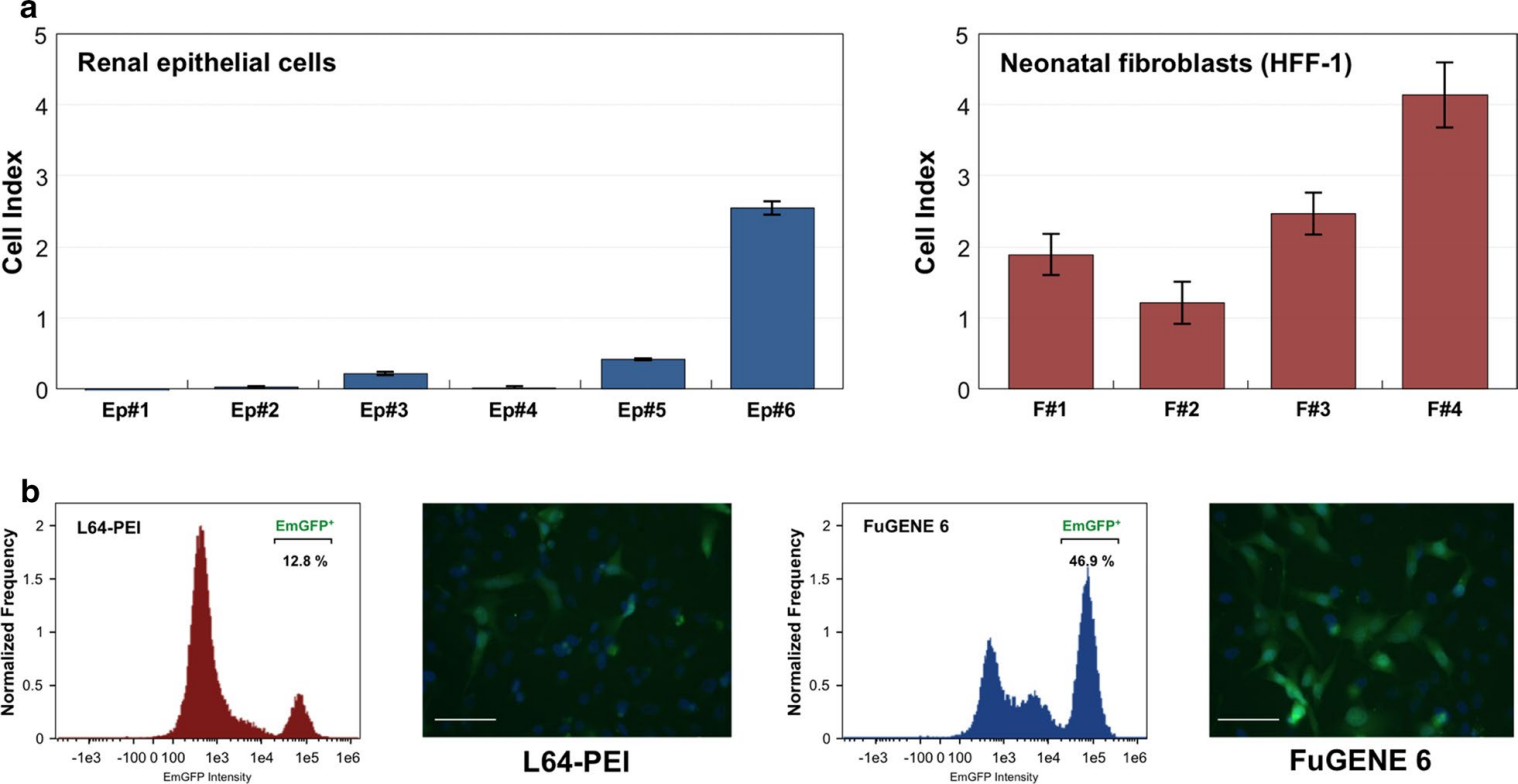
Optimisation of somatic cells culture conditions and transgenes delivery. **a** Cell number was monitored by xCELLigence system. 6000 of epithelial cells or fibroblasts were seeded on one well of the assay plate and maintained in corresponding culture media. Their impact on cell growth was monitored for 72 h and cell index (CI) values at the end of experiment are presented as a mean of three independent counts. **b** Comparison of transfection efficiencies obtained with use of FuGENE 6 reagent and synthesized L64-PEI copolymer. The expression plasmid pCXB-EmGFP was transiently introduced into HeLa cells. Transfection with both reagents was carried out in the presence of FBS and two days later, transfection efficiency was assessed by flow cytometry and fluorescence microscope. *Scale bar* 100 μm

Generation of pluripotent stem cells in conditions that ensure reproducible induction, expansion and their controllable directed differentiation is an indispensable requirement for their therapeutic application. Clinically-relevant iPS cells and differentiated derivatives need to be obtained and maintained in fully defined culture conditions. For this purpose, we aimed to optimize somatic cells culture as well as the step of reprogramming vectors delivery. Different cell culture media were prepared according to compositions shown in Additional files 4 and 5: Tables S4 and S5, and evaluated for their ability to sustain growth of the initial somatic cells. In addition, as control, we tested medium containing foetal bovine serum and commercially available growth media. The impact of medium composition was tested using xCELLigence system that monitors cell proliferation. As shown in Fig. 2a, HFF-1 fibroblasts showed the highest proliferation rate in medium F#3 that contained recombinant albumin, transferrin, EGF, FGF2, PDGF-AB as well as hydrocortisone, sodium selenite and lipid concentrate. For renal epithelial cells, defined medium Ep#5 (containing albumin, transferrin, insulin, EGF, FGF2, PDGF-AB, epinephrine, triiodo-l-thyronine, sodium selenite and chemically defined lipids) provided the highest proliferation rate. However, it needs to be noted that serum-containing media outperformed all abovementioned media in terms of their effect on cell survival and growth.

**Fig. 3:**
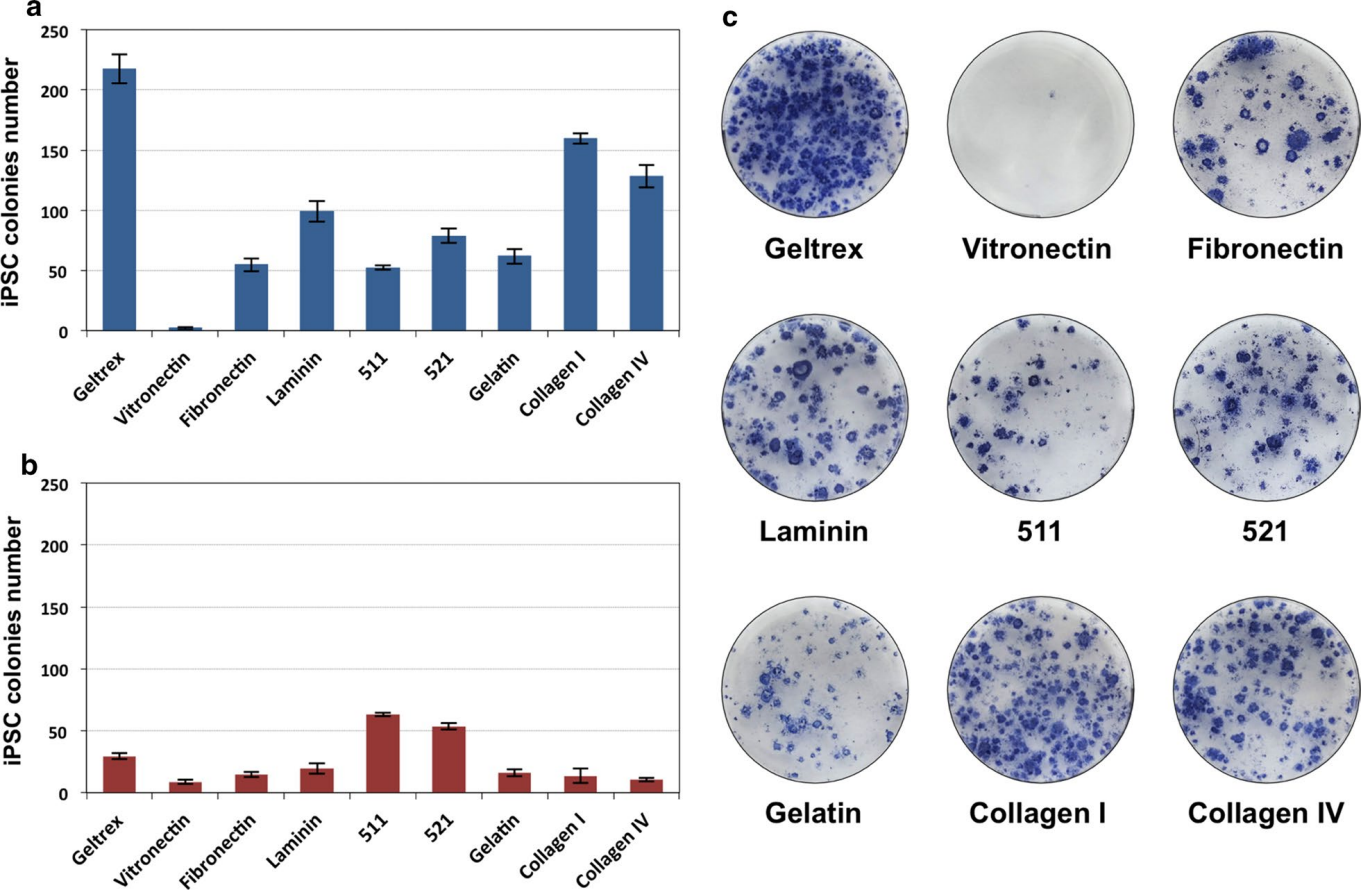
Reprogramming of human fibroblastic and epithelial cells into iPS cells with application of different protein components of the extracellular matrix. **a** Efficiencies of generation of iPS cells obtained from renal epithelial cells. **b** Colony forming efficiencies in the course of fibroblast reprogramming into iPS cells. **c** Exemplary results of alkaline phosphatase staining on iPS cells generated from renal epithelial cells grown on cell culture vessels coated with different proteins and extracellular matrix components

Since previously used transfection reagent, FuGENE 6 (Promega, Madison, WI, USA) has undisclosed composition, we sought the chemically defined alternative. Recently, copolymer of low-weight polyethylenimine (PEI) and Pluronic polymers has been reported as a low-toxic and efficient DNA delivery agent in established cell lines and primary cells [20]. As the L64-PEI (PCM-04) polymer was not commercially available we conducted conjugation of PEI (M_W_ = 1200 Da) to PEG-PPG-PEG block polymer (pluronic L64, M_W_ = 2900 Da) and evaluated its potential as a plasmid delivery carrier. HeLa cells were transfected with pCXB-EmGFP plasmid expressing optimized GFP protein under control of CAGGS promoter using synthesised L64-PEI reagent at 1:3 plasmid to polymer weight ratio. Transfection efficiency was monitored by flow cytometry and epifluorescence microscopy. As shown in Fig. 3b, the number of GFP-positive cells reached 12.8% for cells transfected with L64-PEI, whereas FuGENE 6 transfection reagent provided ~4 times higher efficiency.

In order to better understand the composition of the minimal constituents necessary for the acquisition of pluripotent state by reprogrammed somatic cells, we address the question of the applicability of different protein components of extracellular matrix on the efficiency of pluripotent cells induction. For this purpose, cell culture vessels were coated with murine proteins secreted by Engelbreth-Holm-Swarm (EHS) sarcoma cells (Geltrex, 45 μg/cm^2^), human recombinant Vitronectin (1 μg/cm^2^), porcine gelatin (1 μg/cm^2^), human recombinant Laminin-511 (1 μg/cm^2^), human recombinant Laminin-521 (1 μg/cm^2^), human fibronectin (0.5 μg/cm^2^), human recombinant Collagen I (5 μg/cm^2^), human Collagen IV (5 μg/cm^2^) and fractions of laminins isolated from EHS tumour (1 μg/cm^2^). Induced pluripotent stem cells were derived from adult exfoliated renal epithelial cells and HFF-1 neonatal fibroblasts. These cells were seeded on protein-coated 6-well plates at density 20,000 cells per well (for renal epithelial cells) or 80,000 cells per well (in case of fibroblasts) and transfected with oriP/EBNA-1 episomes as described previously [17]. Reprogramming experiments were carried out for 3–4 weeks till the emergence of compact, tightly packed ESC-like colonies which were fixed with paraformaldehyde and evaluated for the alkaline phosphatase activity. The exemplary phosphatase stainings along with the iPSC colony forming efficiencies in fibroblasts and epithelial cells are presented in Fig. 3. The highest efficiencies of reprogramming of fibroblasts were obtained in case of Laminin-511 (0.08%) and Laminin-521 (0.07%), whereas for human renal epithelial cells the most efficient reprogramming was achieved with Geltrex matrix (1.1%), but also with human, recombinant type I Collagen (0.8%). Altogether, our results confirmed that pluripotency can be induced with application of defined and xeno-free protein components.

Finally, to evaluate polymer utility in derivation of induced pluripotent cells on defined ECM constituents, we plated HFF-1 fibroblasts and urinary epithelial cells on culture vessels coated with recombinant human Collagen I and Laminin-511, respectively. One day later, cells were transfected with 2 μg of episomal plasmids using L64-PEI reagent. The transfection was repeated 24 h later and cells were maintained in TeSR-E7 medium for next 2 weeks. After that, the culture medium was replaced with Essential 8 medium and cells were retained for next 2–3 weeks until the emergence of ESC-like, tightly-packed, clustered colonies. At the end of the experiment, culture plates were fixed with PFA and stained for alkaline phosphatase activity (Additional file 6: Figure S1). The reprogramming efficiencies reached 0.01% for fibroblasts and 0.08% for renal epithelial cells.

Taken together, our results clearly demonstrate that induced pluripotent stem cells can be generated from human somatic cells using fully defined, xeno-free protocols.

### Formation of definitive endoderm in the absence of animal-derived components

**Fig. 4:**
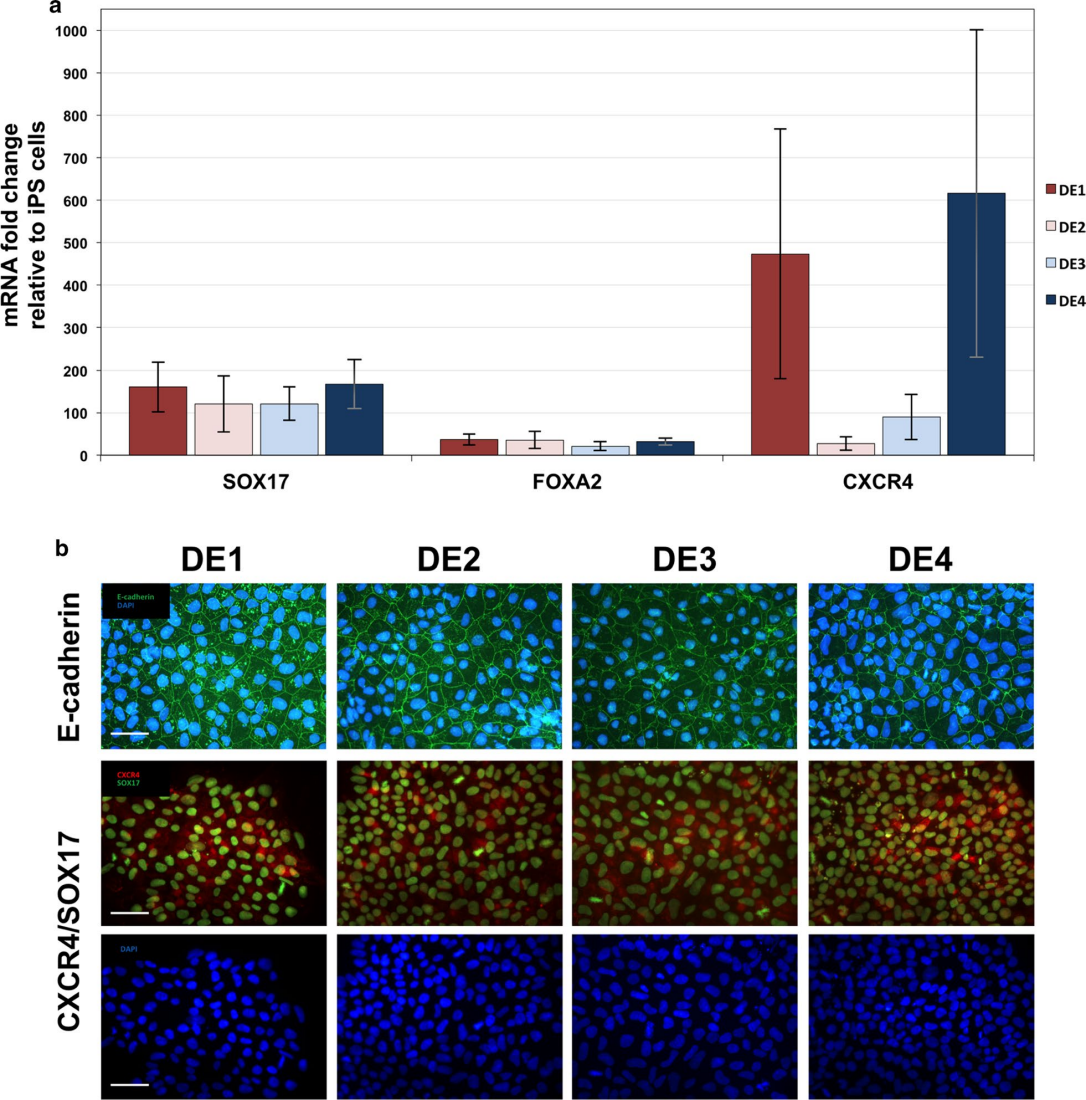
Differentiation of pluripotent cells into definitive endoderm cells. **a** Expression of genes characteristic for definitive endoderm (*CXCR4*, *FOXA2*, *SOX17*) for examined differentiation protocols relative to undifferentiated iPSC samples. For the differentiation experiments cells were maintained in basal medium (RPMI-1640) with Activin A and CHIR99021 for 24 h, and in medium with Activin A alone for the next 48 h. In addition, the media contained: DE1—foetal bovine serum, DE2—human serum, DE3—insulin, transferrin and sodium selenite, DE4—albumin, insulin, transferrin, defined lipids concentrate and ascorbic acid. **b** Results of immunocytochemical analysis of the generated definitive endoderm cells showing cells positive for CXCR4 and SOX17 markers, and clusters of cells displaying E-cadherin expression. Cells were prepared as described in **a**. *Scale bar* 50 µm

iPSC were differentiated into definitive endoderm cells using activators of TGFβ and Wnt signalling pathways. Cells were incubated for 24 h in RPMI1640 basal medium containing 100 ng/mL of Activin A and 3 μM CHIR99021. Next, cells were maintained in culture for 2 days in RPMI1640 medium with Activin A. In addition, these media were supplemented with serum or its replacements to extend lifespan of cells. Four culture media were prepared according to compositions shown in Additional file 7: Table S6. Formulated differentiating media contained 2% of bovine (DE1) or human serum (DE2) or in case of DE3 and DE4 media contained a mixture of various defined additives, deprived from the serum. The influence of these media on the process of definitive endoderm formation was studied by qPCR measurements and immunocytochemical stainings. Levels of mRNA encoding protein markers characteristic for endoderm (*SOX17*, *FOXA2*, *CXCR4*) were analysed and are presented in Fig. 4a, along with raw data included in Additional file 8: Table S7. The highest relative expression of marker genes was observed for FBS-containing medium DE1 and defined medium DE4, that included recombinant albumin, insulin, transferrin as well as ascorbic acid and chemically defined lipids.

Presence of definitive endoderm markers was evaluated on protein level as well, using antibodies against E-cadherin, CXCR4 and SOX17. Results of immunocytochemical analysis are shown in Fig. 4b. For all tested media we detected cells with cytoplasmic presence of CXCR4, SOX17 localized in cell nuclei, as well as clusters of cells positive for E-cadherin.

Collectively, these results indicate that in terms of the efficiency and endodermal markers expression, the definitive endoderm can be formed without animal-derived products at level comparable to culture conditions where serum was included.

### Insulin secretion is enhanced by PDX1 and NKX6.1 induction during IPC progenitors’ maturation

In this study, two strategies for generation of insulin producing cells were coupled. First method involves forced expression of specific transcription factors in differentiating cells. The second approach includes differentiation of initial stem cells by activation or inhibition of certain signalling pathways by small chemical molecules and protein ligands.

**Fig. 5:**
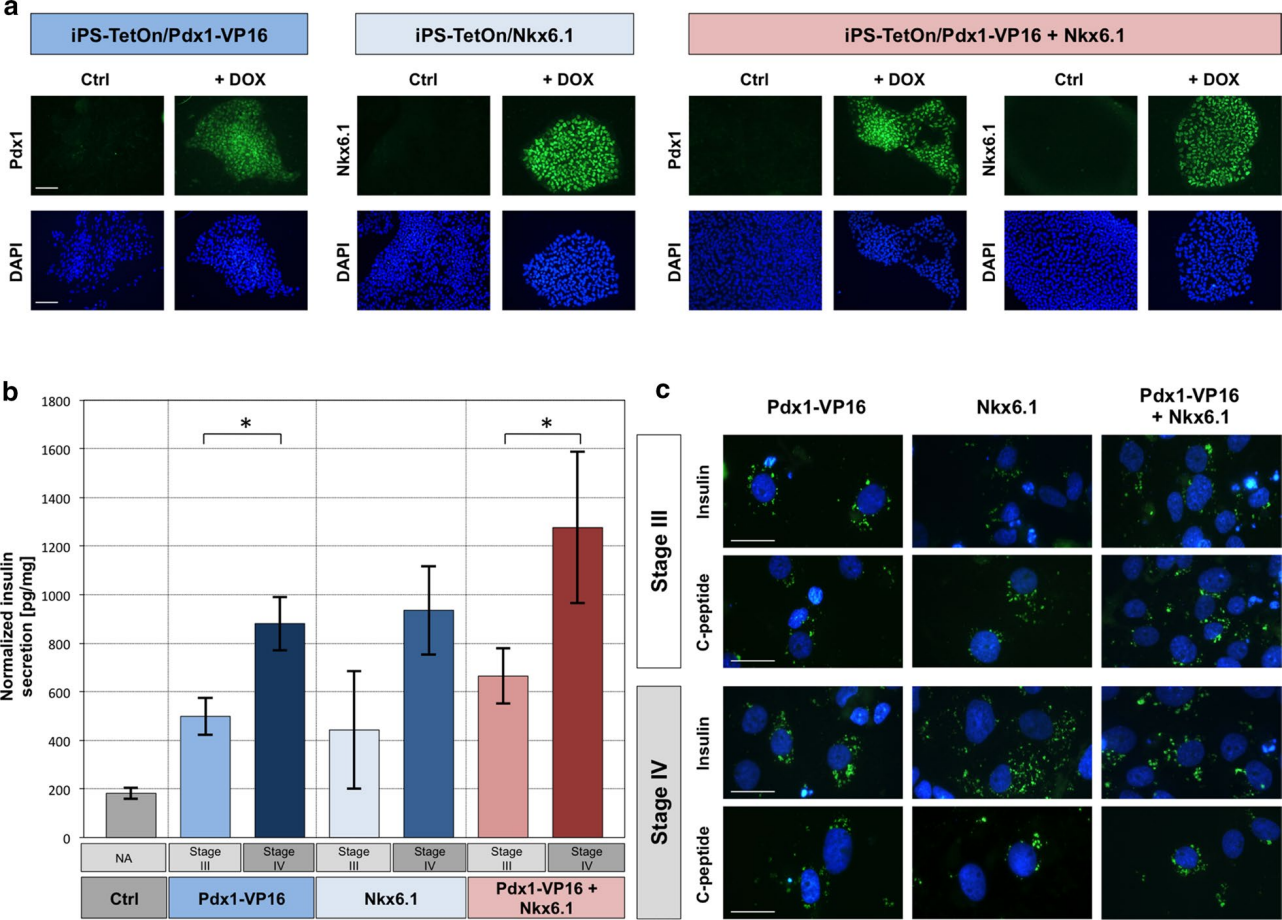
Insulin and C-peptide synthesis by generated insulin producing cells. **a** Doxycycline-regulated Pdx1 and Nkx6.1 expression. iPSC lines with stably integrated Pdx1 and Nkx6.1 transgenes under control of Tre3G promoter were induced by addition of doxycycline and stained with antibodies against Pdx1 and Nkx6.1. The negative control were iPS cells that were not treated with doxycycline. *Scale bar* 100 µm. **b** Insulin secretion in response to 2.5 mM glucose concentration after transgenes induction at selected steps of the differentiation procedure. Results of the ELISA were normalized to the total protein concentration and presented as picograms of produced insulin per milligram of the total protein content of insulin secreting cells. Control includes cells without introduced transgenes. *Asterisk* indicate statistically relevant difference between compared samples. **c** Immunocytochemical detection of insulin and C-peptide production in cells where transgene expression was switched on at indicated stages of differentiation. *Scale bar* 20 µm

To analyse the impact of Pdx1 and Nkx6.1 transcription factors on the process of derivation of insulin producing cells, we generated doxycycline-regulated pluripotent cell lines. For this purpose, iPS cells were transduced with lentiviral vector carrying Tet-On 3G transactivation gene. After the negative selection with G418, these cells were again transduced with lentiviral particles carrying *PDX1*-*VP16* and *NKX6.1* coding sequences under the control of the TRE3G promoter. Following selection with puromycin antibiotic, positive colonies with stably integrated constructs were collected and evaluated for inducible expression by doxycycline. Induced and control samples were analysed by immunocytochemical stainings as shown in Fig. 5a.

Established regulated cell lines were differentiated into definitive endoderm, and further to pancreatic progenitors through the 1 week of incubation in iMEM medium with addition of 1% of B27 or NS21 supplements, 2 μM retinoic acid, 10 μM SB431542, and 1 μM dorsomorphin. For maturation of insulin producing cells, the culture medium was changed to iMEM basal medium supplemented with 1% of NS21, 10 μM forskolin, 10 μM dexamethasone, 10 μM nicotinamide and 5 μM Alk 5 inhibitor II. Cells were maintained in these conditions for 7 days. Expression of *PDX1*-*VP16* and *NKX6.1* transgenes or combination of both was induced by including doxycycline in medium for pancreatic progenitors and/or in medium for pancreatic islets specification as shown on diagram in Fig. 1. To evaluate the ability of obtained cells to secrete insulin, IPC cells were incubate in L15 medium supplemented with 2.5 mM glucose. The secretion of insulin hormone was measured with ELISA assay, normalized for total protein concentration and presented in Fig. 5b. The presence of insulin and C-peptide in established IPC was evaluated by immunocytochemical analysis and shown in Fig. 5c.

**Fig. 6:**
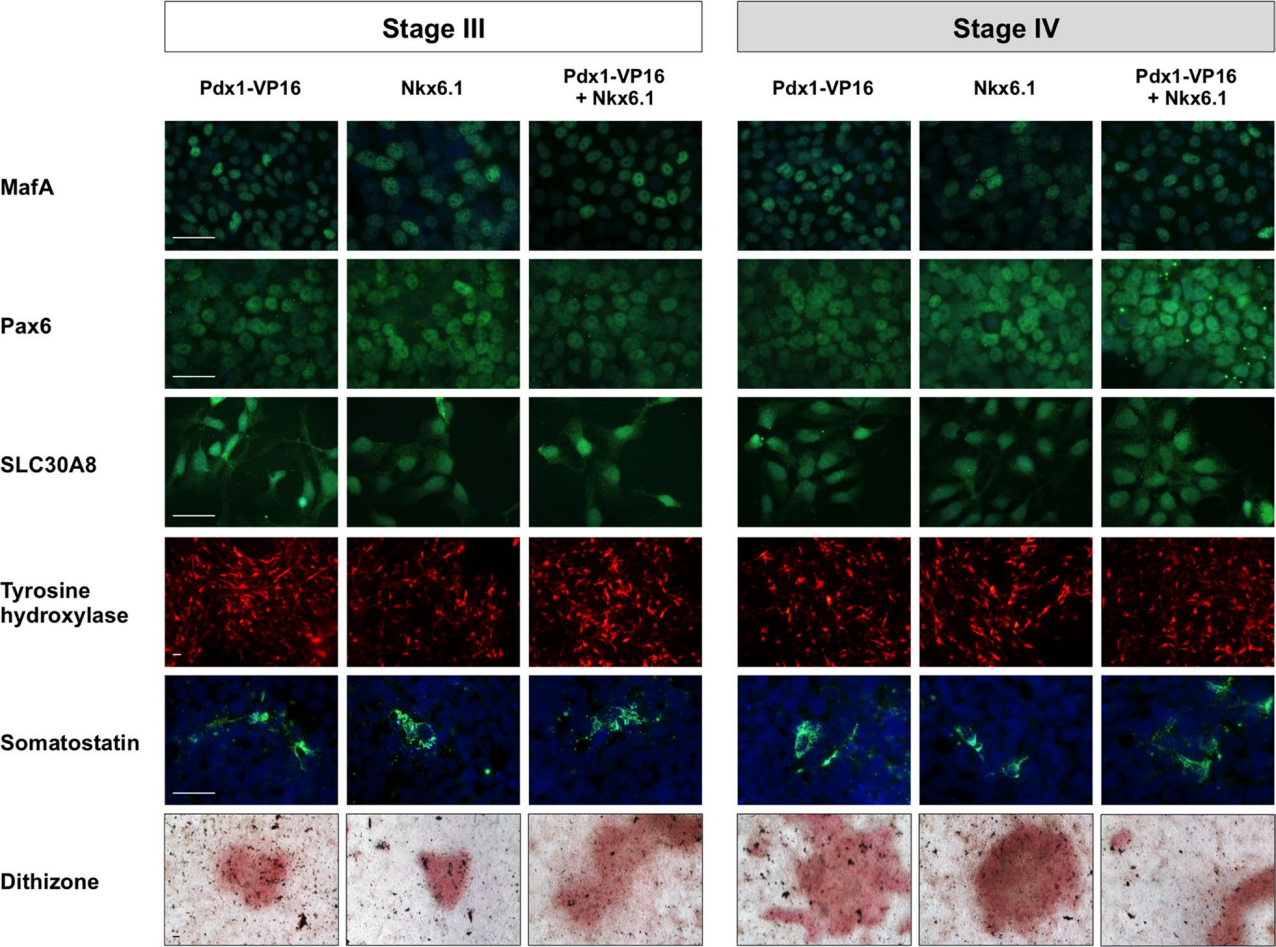
Structural and functional characterisation of established IPCs. Results of immunocytochemical and colorimetric characterisation of generated insulin producing cells. Pluripotent cell lines with stably introduced PDX1-VP16 and NKX6.1 transgenes were differentiated towards insulin producing cells. Transgenes expression was switched on by doxycycline at the stage of IPC progenitors or maturation of insulin producing cells. Differentiated cells were immunophenotyped with antibodies against MafA, Pax6, SLC30A8, somatostatin, tyrosine hydroxylase (all except TH counterstained with DAPI), and stained with dithizone. The images represent selected clusters of positive cells in the population of differentiated cells. *Scale bar* 100 µm

In order to further determine the identity of differentiated cells, we performed immunocytochemical analysis using antibodies characteristic for β-cells and pancreatic lineage. iPSC lines expressing PDX1-VP16, NKX6.1 and combination of both factors from the doxycycline-regulated promoter were differentiated into insulin producing cells. Expression of transgenes was induced either on the stage of generation of IPC progenitors or during maturation of insulin producing cells. Results of experiments shown in Fig. 6 revealed in all studied samples the presence of cell clusters positive for MafA, Pax6, SLC30A8 and Tyrosine hydroxylase. With use of Somatostatin-specific antibodies we could detect single hormone expressing cells among differentiated cell populations. In addition to SLC30A8 protein detection in ICC assay, we performed stainings with dithizone, a zinc-chelating agent, which indicated the presence of functional zinc transporter ZnT8 in obtained insulin producing cells.

Altogether, generated cells were shown to secrete insulin into the culture medium and displayed molecular markers of pancreatic cells. However, the highest insulin concentration was detected in case of cells with transcription factors induction at stage IV (maturation of Insulin Producing Cells). Furthermore, cells expressing both PDX1-VP16 and NKX6.1 secreted higher level of the hormone, regardless the induction time point.

## Discussion

Regulatory issues related to cell-based therapies are getting broadened to acknowledge treatments with use of stem cells and products of their differentiation. Although few clinical trials based on application of differentiated embryonic stem cells have been approved, there are still safety concerns that need to be thoroughly addressed. These include among others, derivation of pluripotent stem cells, identity and homogeneity of clinically-relevant cells and their immunogenic potential. The last issue can be resolved by usage of either patient-specific or allogenic, HLA-matched iPSC further differentiated into required cells. Establishment of fully defined conditions of iPSC generation and differentiation would settle some of the abovementioned concerns and therefore be a step towards putting these cells closer to clinical applications.

In this work we made an attempt to generate induced pluripotent cells and differentiate them into insulin producing cells. Development of reproducible protocols for differentiation of iPSC into specified multipotent progenitors and terminally differentiated cells remains challenging. In particular, the application of components originating from animals for manufacturing of cells for therapeutic use pose several problems due to batch-to-batch variation and the inherited inconsistency of animal-derived products. Additional concerns involve the contamination with adventitious agents that may result in animal-to-human pathogen transmission in the course of therapy. As a consequence, there is a high demand from regulatory bodies such as Food and Drug Administration (FDA) and the European Medicines Agency (EMA) to put restrictions on the application of animal products in cell-based therapeutics [22, 23]. For this reason, our aim was to establish efficient and reliable protocols based on defined components. In the first part of our work, we evaluated the ability of different extracellular matrix (ECM) constituents to support induction of pluripotency in reprogrammed somatic cells. We tested recombinant or xeno-free Vitronectin, Fibronectin, Laminins and Collagens, and compared them with undefined matrices. Although, some of these proteins were reported to maintain pluripotency markers in already established iPSC or ESC lines [24–28], they were not evaluated in the systematic manner for induction of pluripotency.

Our results demonstrated that iPSC colonies could be obtained on all tested matrix components, including precisely defined protein constituents, although with varying efficiencies. Neonatal fibroblasts were reprogrammed with highest efficiencies on recombinant Laminin-511 and Laminin-521. These proteins were detected on human embryonic stem cells and it is presumed that they play a role in maintaining intrinsic properties of pluripotent stem cells [25]. In particular, the globular domain of laminins interact with α6β1 integrins present on cell membrane. This activates PIK3CA/AKT signalling pathway, which influence numerous cytophysiological processes including their self-renewal ability [29, 30]. Although its precise mechanisms remains elusive, the interaction responsible for cell proliferation may trigger signalling events that influence transcriptional status and reprogramming rate of fibroblasts [26, 31]. Integrins serve as connectors for cell-to-cell and cell-to-ECM interactions. These heterodimeric transmembrane receptors consists of α and β subunits, which can make up to 24 different integrin combinations. Different receptor variants have affinities for specific ECM components; for instance, integrin αVβ5 interacts with vitronectin, α5β1 with fibronectin and α6β1 integrin binds to laminins [32]. On the other hand, there is also an interplay between iPSC generation and expression of particular integrin receptors, as the Oct3/4 and Sox2 transcription factors bind to regulatory regions of α6 and β1 subunits coding sequences [33, 34], and during reprogramming of fibroblasts into iPS cells, the expression of α6 subunit is upregulated within 3 days after fibroblast transduction with retroviruses carrying reprogramming factors [35]. Conversion of renal epithelial cells into iPSC was the most effective on Geltrex matrix and recombinant Collagen I. Geltrex is a compound matrix comprising different proteins secreted by EHS sarcoma cells including laminins, entactin and collagens. Notably, Collagen type I and IV is thought to influence stem cells self-renewal, as their binding to integrin α2β1 and DDR1 receptor [36] activates Bmi-1 protein involved in cell proliferation [37].

In our attempt to establish fully defined conditions for generation of induced pluripotent cells we used copolymer of low-weight polyethylenimine and PEG-PPG-PEG block polymer (pluronic L64). This compound was developed by Wang and co-workers [20] for DNA delivery applications. In our studies we successfully delivered episomal reprogramming plasmids into human cells of epithelial and fibroblastic origin achieving iPSC formation rate comparable to the previously used commercially available transfection reagent [17].

The definitive endoderm (DE) is formed during gastrulation and participates in morphogenesis of digestive tract including stomach, small and large intestine, liver and pancreas, and it can also contribute to development of lungs, thymus and thyroid. DE formation is induced by activation of TGFβ and Wnt signalling pathways. In the in vitro experimental setup, the presence of Activin A and Wnt3a or inhibitors of GSK3β is essential for the establishment of definitive endoderm. In most of available protocols varying concentrations of FBS were used as the absence of serum was reported to severely restrict the induction of DE or results in poor cell survival [38–40]. In this work we sought to replace FBS in order to establish defined conditions for DE cells generation. Our data clearly indicate that a combination of recombinant albumin, insulin, transferrin as well as lipid concentrate and ascorbic acid is as efficient in definitive endoderm induction as FBS or human serum and is sufficient to substitute them.

Pancreas develops from endoderm of the anterior intestine upon signalling through the specific receptors, which in turn activates the network of transcription factors necessary for differentiation of endocrine cells. The pancreas is formed from the fusion of the dorsal and ventral pancreatic ducts. The pancreatic buds appear at about 25th day of human gestation arising from the distal foregut endoderm. Development of insulin producing cells begins at the end of the embryonic period around day 56 post-conception, when the number of insulin-positive cells starts to gradually increase. The Langerhan’s islets are formed in the first trimester of the pregnancy about 12 week of the gestation [41]. All of these events are triggered by nearby cells and tightly controlled. Signals driving formation of dorsal bud involve release of FGF2 and Activin β from the notochord cells, and they are implicated in inhibition of the Hedgehog pathway in epithelial cells forming the dorsal bud (reviewed in [42]). During endocrine cell development, Notch signalling plays an important role in maintaining the balance between the expansion of progenitor cells and their differentiation into mature cell types. Its downstream target, Hes-1 influences transcription of Ngn3, which is a key activator of endocrine pancreas commitment [43]. Pdx1 is one of the first transcription factors expressed in foregut endoderm and its requirement for pancreas development is evident from the knock-out studies that resulted in the absence of pancreas at birth [44]. The transcription factor is essential for the development of insulin producing cells, as Pdx1 regulates insulin production in glucose-dependant manner. The mRNA level of Pdx1 declines in fully developed endocrine cells, and the factor expression is restricted to mature β-cells [45]. Nkx6.1 is another transcription factor that was found to be specific for adult insulin producing cells [46]. This factor controls gene regulatory network necessary for establishment of beta cell identity. In addition, Nkx6.1 is involved in lineage specification in early pancreatic progenitor tissue as it has been found to repress the commitment of the endocrine progenitors to non-beta cells [15].

In this work, we analysed the influence of induced expression of the two abovementioned factors, Pdx1 and Nkx6.1 and their concurrent activation on the process of differentiation of human pluripotent stem cells towards insulin producing cells. The effect of Pdx1 exogenous expression on differentiation or transdifferentiation processes of various cells into IPCs was previously reported, either as a sole introduced factor [47–49] or in combination with NeuroD1 and MafA [50], but not with Nkx6.1. Our results demonstrate for the first time that differences in insulin secretion depend on the differentiation stage at which transcription factors expression was induced. Notably, the highest insulin production was obtained in case of simultaneous activation of both transgenes throughout maturation of insulin producing cells. Analysis of MafA PAX6, SLC30A8, Tyrosine Hydroxylase, Somatostatin, as well as functional assay of ZnT8, did provide evident insight into the mechanism responsible for the difference between stage III and stage IV cells ability to produce insulin. The selected transcription factors play critical role in development and proper functioning of pancreas since mutations in genes encoding these factors or alterations in their expression are associated with diseases and multitude of developmental abnormalities [51–53]. Pdx1 and Nkx6.1 control expression of a number of pancreas-related genes [15, 54]. Therefore, it is difficult to identify the precise molecular mechanisms that are responsible for the observed results. However, one could hypothesise that the overexpression of these factors at the stage of maturation of insulin producing cells can interfere with signalling pathways responsible for establishment of such cells. As in the process of embryogenesis, varying expression of certain genes leads to the formation of all types of hormone-releasing islet cells [55, 56], one may conclude that the forced expression of Pdx1 and Nkx6.1 can shift the developmental balance toward the increased formation of insulin producing cells. Moreover, it seems that simultaneous expression of both factors enhances the emergence of beta cell-like cells, as the obtained cells share more characteristic features with cells of native pancreatic islets, particularly in the level of insulin secretion.

## Conclusions

In this work, insulin producing cells were generated from induced pluripotent stem cells in defined culture conditions. Stem cells were differentiated by culture in media containing small chemical molecules and recombinant proteins with concurrent overexpression of specific transcription factors at different steps of IPC formation. Our results indicate that it is possible to obtain insulin producing cells in fully defined culture setup that is free from animal-derived components, which is essential for the prospective clinical applications. In addition, we demonstrated that induced expression of *PDX1* and *NKX6.1* genes, which encode transcription factors indispensable for pancreatic development, at the stage of maturation of IPSC enhances production of insulin. Furthermore, the concurrent induction of both factors in the course of the differentiation process results in highest level of secretion of the hormone.

## Additional files

**Additional file 1: Table S1.** List of primers used for cloning and sequencing.

**Additional file 2: Table S2.** Antibodies used for immunocytochemical analysis.

**Additional file 3: Table S3.** List of primers used for quantitative real-time PCR reactions.

**Additional file 4: Table S4.** Composition of media used for culture of renal epithelial cells.

**Additional file 5: Table S5.** Composition of media used for culture of renal epithelial cells.

**Additional file 6: Figure S1.** Derivation of iPS cells in defined culture conditions.

**Additional file 7: Table S6.** Composition of media used for derivation of the definitive endoderm from iPS cells.

**Additional file 8: Table S7.** Comparison of relative SOX17, FOXA2 and CXCR4 expression levels in definitive endoderm cells by means of quantitative RT-PCR.

## Abbreviations

BCIP: 5-bromo-4-chloro-3-indolyl phosphate
BMI-1: polycomb group RING finger protein 4
CI: cell index
C_T_: threshold cycle value
DE: definitive endoderm
DMEM: Dulbecco’s modified eagle medium
DMSO: dimethyl sulfoxide
ECM: extracellular matrix
EDTA: ethylenediaminetetraacetic acid
EGF: epidermal growth factor
EHS: Engelbreth-Holm-Swarm murine sarcoma
EMA: European Medicines Agency
EMT: epithelial-mesenchymal transition
FBS: foetal bovine serum
FDA: Food and Drug Administration
FGF2: fibroblast growth factor 2
GFP: green fluorescent protein
GSK3β: glycogen synthase kinase 3 beta
hESC: human embryonic stem cells
HLA: human leukocyte antigen system
ICC: immunocytochemistry
iMEM: improved minimum essential medium
IPC: insulin producing cells
iPSC: induced pluripotent stem cells
M_W_: molecular weight
NBT: nitro-blue tetrazolium
PBS: phosphate-buffered saline
PCR: polymerase chain reaction
PDGF-AB: platelet-derived growth factor AB chains
PEI: polyethylenimine
PFA: paraformaldehyde
PIK3CA/AKT: phosphatidylinositol-4,5-bisphosphate 3-kinase/protein kinase B
qPCR: quantitative polymerase chain reaction
REGM: renal cell growth medium
RPMI1640: Roswell Park Memorial Institute 1640 Medium
RTCA: real-time cell analyzer
SEM: standard error of the mean
T1D: type 1 diabetes
TBS-T: tris-buffered saline, tween-20
TGFβ: transforming growth factor β
TH: tyrosine hydroxylase

## References

[1] Maahs DM, West NA, Lawrence JM, Mayer-Davis EJ (2010). Epidemiology of type 1 diabetes. Endocrinol Metab Clin North Am.

[2] Johnson PRV, Jones KE (2012). Pancreatic islet transplantation. Semin Pediatr Surg.

[3] Takahashi K, Yamanaka S (2006). Induction of pluripotent stem cells from mouse embryonic and adult fibroblast cultures by defined factors. Cell.

[4] Mallon BS, Hamilton RS, Kozhich OA, Johnson KR, Fann YC, Rao MS (2014). Comparison of the molecular profiles of human embryonic and induced pluripotent stem cells of isogenic origin. Stem Cell Res.

[5] Bilic J, Belmonte JCI (2012). Concise review: induced pluripotent stem cells versus embryonic stem cells: close enough or yet too far apart?. Stem Cells.

[6] Zhao J, Jiang W, Sun C, Hou C, Yang X, Gao J (2013). Induced pluripotent stem cells: origins, applications, and future perspectives. J Zhejiang Univ Sci B.

[7] Hosoya M, Kunisada Y, Kurisaki A, Asashima M (2012). Induction of differentiation of undifferentiated cells into pancreatic beta cells in vertebrates. Int J Dev Biol.

[8] Hosoya M (2012). Preparation of pancreatic β-cells from human iPS cells with small molecules. Islets.

[9] Best M, Carroll M, Hanley NA, Hanley KP (2008). Embryonic stem cells to beta-cells by understanding pancreas development. Mol Cell Endocrinol.

[10] D’Amour KA, Bang AG, Eliazer S, Kelly OG, Agulnick AD, Smart NG (2006). Production of pancreatic hormone–expressing endocrine cells from human embryonic stem cells. Nat Biotechnol.

[11] Chen S, Borowiak M, Fox JL, Maehr R, Osafune K, Davidow L (2009). A small molecule that directs differentiation of human ESCs into the pancreatic lineage. Nat Chem Biol.

[12] Thatava T, Nelson TJ, Edukulla R, Sakuma T, Ohmine S, Tonne JM (2011). Indolactam V/GLP-1-mediated differentiation of human iPS cells into glucose-responsive insulin-secreting progeny. Gene Ther.

[13] Kunisada Y, Tsubooka-Yamazoe N, Shoji M, Hosoya M (2012). Small molecules induce efficient differentiation into insulin-producing cells from human induced pluripotent stem cells. Stem Cell Res.

[14] Johnson JD, Ahmed NT, Luciani DS, Han Z, Tran H, Fujita J (2003). Increased islet apoptosis in Pdx1 ± mice. J Clin Invest.

[15] Schaffer AE, Taylor BL, Benthuysen JR, Liu J, Thorel F, Yuan W (2013). Nkx6.1 controls a gene regulatory network required for establishing and maintaining pancreatic beta cell identity. PLoS Genet.

[16] Hashimoto H, Kamisako T, Kagawa T, Haraguchi S, Yagoto M, Takahashi R (2015). Expression of pancreatic and duodenal homeobox1 (PDX1) protein in the interior and exterior regions of the intestine, revealed by development and analysis of *Pdx1* knockout mice. Lab Anim Res.

[17] Drozd AM, Walczak MP, Piaskowski S, Stoczynska-Fidelus E, Rieske P, Grzela DP (2015). Generation of human iPSCs from cells of fibroblastic and epithelial origin by means of the oriP/EBNA-1 episomal reprogramming system. Stem Cell Res Ther.

[18] Zhou T, Benda C, Dunzinger S, Huang Y, Ho JC, Yang J (2012). Generation of human induced pluripotent stem cells from urine samples. Nat Protoc.

[19] Cho KC, Choi SH, Park TG (2006). Low molecular weight PEI conjugated pluronic copolymer: useful additive for enhancing gene transfection efficiency. Macromol Res.

[20] Wang M, Lu P, Wu B, Tucker JD, Cloer C, Lu Q (2012). High efficiency and low toxicity of polyethyleneimine modified pluronics (PEI–pluronic) as gene delivery carriers in cell culture and dystrophic mdx mice. J Mater Chem.

[21] Pfaffl MW (2001). A new mathematical model for relative quantification in real-time RT-PCR. Nucleic Acids Res.

[22] Oikonomopoulos A, van Deen WK, Manansala A-R, Lacey PN, Tomakili TA, Ziman A (2015). Optimization of human mesenchymal stem cell manufacturing: the effects of animal/xeno-free media. Sci Rep.

[23] Desai N, Rambhia P, Gishto A (2015). Human embryonic stem cell cultivation: historical perspective and evolution of xeno-free culture systems. Reprod Biol Endocrinol.

[24] Kaini RR, Shen-Gunther J, Cleland JM, Greene WA, Wang H-C (2015). Recombinant xeno-free vitronectin supports self-renewal and pluripotency in protein-induced pluripotent stem cells. Tissue Eng Part C Methods.

[25] Miyazaki T, Futaki S, Hasegawa K, Kawasaki M, Sanzen N, Hayashi M (2008). Recombinant human laminin isoforms can support the undifferentiated growth of human embryonic stem cells. Biochem Biophys Res Commun.

[26] Rodin S, Domogatskaya A, Ström S, Hansson EM, Chien KR, Inzunza J (2010). Long-term self-renewal of human pluripotent stem cells on human recombinant laminin-511. Nat Biotechnol.

[27] Chen G, Gulbranson DR, Hou Z, Bolin JM, Ruotti V, Probasco MD (2011). Chemically defined conditions for human iPS cell derivation and culture. Nat Methods.

[28] Lambshead JW, Meagher L, O’Brien C, Laslett AL (2013). Defining synthetic surfaces for human pluripotent stem cell culture. Cell Regen.

[29] Rodin S, Antonsson L, Niaudet C, Simonson OE, Salmela E, Hansson EM (2014). Clonal culturing of human embryonic stem cells on laminin-521/E-cadherin matrix in defined and xeno-free environment. Nat Commun.

[30] Lu HF, Chai C, Lim TC, Leong MF, Lim JK, Gao S (2014). A defined xeno-free and feeder-free culture system for the derivation, expansion and direct differentiation of transgene-free patient-specific induced pluripotent stem cells. Biomaterials.

[31] Domogatskaya A, Rodin S, Boutaud A, Tryggvason K (2008). Laminin-511 but Not -332, -111, or -411 enables mouse embryonic stem cell self-renewal in vitro. Stem Cells.

[32] Hynes RO (1992). Integrins: versatility, modulation, and signaling in cell adhesion. Cell.

[33] Boyer LA, Lee TI, Cole MF, Johnstone SE, Levine SS, Zucker JP (2005). Core transcriptional regulatory circuitry in human embryonic stem cells. Cell.

[34] Lodato MA, Ng CW, Wamstad JA, Cheng AW, Thai KK, Fraenkel E (2013). SOX2 Co-occupies distal enhancer elements with distinct POU factors in ESCs and NPCs to specify cell state. PLoS Genet.

[35] Villa-Diaz LG, Kim JK, Laperle A, Palecek SP, Krebsbach PH (2016). Inhibition of focal adhesion kinase signaling by integrin α6β1 supports human pluripotent stem cell self-renewal. Stem Cells Dayt Ohio.

[36] Staudinger LA, Spano SJ, Lee W, Coelho N, Rajshankar D, Bendeck MP (2013). Interactions between the discoidin domain receptor 1 and β1 integrin regulate attachment to collagen. Biol Open.

[37] Suh HN, Han HJ (2011). Collagen I regulates the self-renewal of mouse embryonic stem cells through α2β1 integrin- and DDR1-dependent Bmi-1. J Cell Physiol.

[38] Tada S, Era T, Furusawa C, Sakurai H, Nishikawa S, Kinoshita M (2005). Characterization of mesendoderm: a diverging point of the definitive endoderm and mesoderm in embryonic stem cell differentiation culture. Dev Camb Engl.

[39] Morrison GM, Oikonomopoulou I, Migueles RP, Soneji S, Livigni A, Enver T (2008). Anterior definitive endoderm from ESCs reveals a role for FGF signaling. Cell Stem Cell.

[40] Sui L, Bouwens L, Mfopou JK (2013). Signaling pathways during maintenance and definitive endoderm differentiation of embryonic stem cells. Int J Dev Biol.

[41] Piper K, Ball SG, Turnpenny LW, Brickwood S, Wilson DI, Hanley NA (2002). Beta-cell differentiation during human development does not rely on nestin-positive precursors: implications for stem cell-derived replacement therapy. Diabetologia.

[42] Hebrok M (2003). Hedgehog signaling in pancreas development. Mech Dev.

[43] Jensen J, Pedersen EE, Galante P, Hald J, Heller RS, Ishibashi M (2000). Control of endodermal endocrine development by Hes-1. Nat Genet.

[44] Offield MF, Jetton TL, Labosky PA, Ray M, Stein RW, Magnuson MA (1996). PDX-1 is required for pancreatic outgrowth and differentiation of the rostral duodenum. Development.

[45] Ahlgren U, Jonsson J, Jonsson L, Simu K, Edlund H (1998). β-Cell-specific inactivation of the mouseIpf1/Pdx1 gene results in loss of the β-cell phenotype and maturity onset diabetes. Genes Dev.

[46] Jensen J, Serup P, Karlsen C, Nielsen TF, Madsen OD (1996). mRNA profiling of rat islet tumors reveals nkx 6.1 as a beta-cell-specific homeodomain transcription factor. J Biol Chem.

[47] Boroujeni ZN, Aleyasin A (2013). Insulin producing cells established using non-integrated lentiviral vector harboring PDX1 gene. World J Stem Cells.

[48] Delisle JC, Martignat L, Dubreil L, Saï P, Bach J-M, Louzier V (2009). Pdx-1 or Pdx-1-VP16 protein transduction induces β-cell gene expression in liver-stem WB cells. BMC Res Notes.

[49] Cao L-Z, Tang D-Q, Horb ME, Li S-W, Yang L-J (2004). High glucose is necessary for complete maturation of Pdx1-VP16—expressing hepatic cells into functional insulin-producing cells. Diabetes.

[50] Qing-Song G, Ming-Yan Z, Lei W, Xiang-Jun F, Yu-Hua L, Zhi-Wei W (2012). Combined transfection of the three transcriptional factors, PDX-1, NeuroD1, and MafA, causes differentiation of bone marrow mesenchymal stem cells into insulin-producing cells. J Diabetes Res J Diabetes Res.

[51] Taylor BL, Liu F-F, Sander M (2013). Nkx6.1 is essential for maintaining the functional state of pancreatic beta cells. Cell Rep.

[52] Fujimoto K, Polonsky KS (2009). Pdx1 and other factors that regulate pancreatic beta-cell survival. Diabetes Obes Metab.

[53] Jennings RE, Berry AA, Strutt JP, Gerrard DT, Hanley NA (2015). Human pancreas development. Development.

[54] Qin Y, Xiao L, Zhan XB, Zhou HX (2015). Pdxl and its role in activating Ngn3 and Pax6 to induce differentiation of iPSCs into islet β cells. Genet Mol Res GMR.

[55] van der Meulen T, Huising MO (2015). The role of transcription factors in the transdifferentiation of pancreatic islet cells. J Mol Endocrinol.

[56] Murtaugh LC (2007). Pancreas and beta-cell development: from the actual to the possible. Development..

