# Supplementary material for: Directed differentiation of human iPSC into insulin producing cells is improved by induced expression of PDX1 and NKX6.1 factors in IPC progenitors
Source: J Transl Med. 2016 Dec 20;14:341. doi: 10.1186/s12967-016-1097-0 (PMC5168869; doi:10.1186/s12967-016-1097-0)
Supplement: Supplementary file 1 — Additional file 1: Table S1. List of primers used for cloning and sequencing. [file 12967_2016_1097_MOESM1_ESM.pdf]

Table S1. List of primers used for cloning and sequencing.

Primer	Sequence (5'→3')
**FP attB1**	GGG GAC AAG TTT GTA CAA AAA AGC AGC GTA TGG TGA GCA AGG GCG AGG A
**FP attB2**	GGG GAC CAC TTT GTA CAA GAA AGC TGG GTC TTG TAC AGC TCG TCC ATG CCG
**Gateway 1**	CAC ATT ATA CGA GCC GGA AGC AT
**Gateway 2**	CAG TGT GCC GGT CTC CGT TAT CG
**M13 Fwd (−20)**	GTA AAA CGA CGG CCA G
**M13 Rev**	CAG GAA ACA GCT ATG AC
**Nkx6.1 attB1**	GGG GAC AAG TTT GTA CAA AAA AGC AGG CTA CCA TGG AGG GCA CCC GGC AGA GCG CAT TCC TG
**Nkx6.1 attB2**	GGG GAC CAC TTT GTA CAA GAA AGC TGG GTT TCA GGA TGA GCT CTC CGG CTC GGA CGC GTG C
**Pdx1-VP16 attB1**	GGG GAC AAG TTT GTA CAA AAA AGC AGC GTA TGA ACG GCG AGG AGC AGT ACT ACG CG
**Pdx1-VP16 attB2**	GGG GAC CAC TTT GTA CAA GAA AGC TGG GTT CGT GGT TCC TGC GGC CG
**pLVX-TRE3G Fwd**	CAG ATC GCC TGG AGC AAT TC
**pLVX-TRE3G Rev**	GCT CCA GAC TGC CTT GGG AA
