# Supplementary material for: Directed differentiation of human iPSC into insulin producing cells is improved by induced expression of PDX1 and NKX6.1 factors in IPC progenitors
Source: J Transl Med. 2016 Dec 20;14:341. doi: 10.1186/s12967-016-1097-0 (PMC5168869; doi:10.1186/s12967-016-1097-0)
Supplement: Supplementary file 2 — Additional file 2: Table S2. Antibodies used for immunocytochemical analysis. [file 12967_2016_1097_MOESM2_ESM.pdf]

**Table S2.** Antibodies used for immunocytochemical analysis

| Target | Host | Manufacturer | Catalogue number | Working dilution |
|---|---|---|---|---|
| **C-Peptide** | rabbit | Cell Signalling | 4593S | 1:100 |
| **CXCR4** | mouse | Santa Cruz Biotechnology | sc-12764 | 1:200 |
| **E-cadherin** | rabbit | Santa Cruz Biotechnology | sc- 7870 | 1:200 |
| **Insulin** | rabbit | Cell Signalling | 4590S | 1:100 |
| **Mouse IgG,** (Alexa 594 conjugated) | donkey | Life Technologies | A21203 | 1:500 |
| **NKX6.1** | rabbit | Abcam | ab90706 | 1:200 |
| **PDX1** | rabbit | Abcam | ab47267 | 1:200 |
| **Rabbit IgG,** (Alexa 488 conjugated) | donkey | Life Technologies | A21206 | 1:500 |
| **SOX17** | rabbit | Millipore | 09-038 | 1:100 |
| **MafA** | rabbit | Pierce | PA5-26450 | 1:100 |
| **Pax6** | rabbit | Abcam | ab5790 | 1:100 |
| **SLC30A8** | rabbit | Sigma-Aldrich | SAB3500538 | 1:100 |
| **Tyrosine hydroxylase** | mouse | Santa Cruz Biotechnology | sc-25269 | 1:250 |
| **Somatostatin** | rabbit | Santa Cruz Biotechnology | sc-13099 | 1:200 |
