# Supplementary material for: Directed differentiation of human iPSC into insulin producing cells is improved by induced expression of PDX1 and NKX6.1 factors in IPC progenitors
Source: J Transl Med. 2016 Dec 20;14:341. doi: 10.1186/s12967-016-1097-0 (PMC5168869; doi:10.1186/s12967-016-1097-0)
Supplement: Supplementary file 3 — Additional file 3: Table S3. List of primers used for quantitative real-time PCR reactions. [file 12967_2016_1097_MOESM3_ESM.pdf]

Table S3. List of primers used for quantitative real-time PCR reactions.

Primer	Sequence (5'→3')	Amplicon size (bp)
**18S rRNA Fwd**	GTA ACC CGT TGA ACC CCA TT	151
**18S rRNA Rev**	CCA TCC AAT CGG TAG TAG CG	
**CXCR4 Fwd**	TCT ACA GCA GTG TCC TCA TCC TGG CC	150
**CXCR4 Rev**	GAA TAG TCA GCA GGA GGG CAG GGA T	
**FOXA2 Fwd**	CGA GTT GAG CCT GTG AGG CCT GG	150
**FOXA2 Rev**	ACT CGG GCA ACA TGT TCG AGA ACG G	
**SOX17 Fwd**	ACC TAC AGC TAC GCG CAG GTC TCG	151
**SOX17 Rev**	TCG CGC CGT AGT ACA CGT GAA GGG	
