# Supplementary material for: Directed differentiation of human iPSC into insulin producing cells is improved by induced expression of PDX1 and NKX6.1 factors in IPC progenitors
Source: J Transl Med. 2016 Dec 20;14:341. doi: 10.1186/s12967-016-1097-0 (PMC5168869; doi:10.1186/s12967-016-1097-0)
Supplement: Supplementary file 5 — Additional file 5: Table S5. Composition of media used for culture of renal epithelial cells. [file 12967_2016_1097_MOESM5_ESM.pdf]

Table S5. Composition of media used for culture of renal epithelial cells.

**F#1**	DMEM, human recombinant Insulin 10 μg/mL, human recombinant Transferrin 10 μg/mL, sodium selenite 20 nM
**F#2**	DMEM, human recombinant Albumin 1 mg/mL, human recombinant Insulin 10 μg/mL, human recombinant Transferrin 10 μg/mL, sodium selenite 20 nM
**F#3**	DMEM, human recombinant Albumin 1 mg/mL, EGF 10 ng/mL, Fgf2 5 ng/mL, PDGF-AB 5 ng/mL, hydrocortisone 36 ng/mL, human recombinant Insulin 10 μg/mL, human recombinant Transferrin 10 μg/mL, sodium selenite 20 nM, chemically defined lipid concentrate 1:50 dilution
**F#4**	DMEM, Foetal Bovine Serum 10%
