# Supplementary material for: Directed differentiation of human iPSC into insulin producing cells is improved by induced expression of PDX1 and NKX6.1 factors in IPC progenitors
Source: J Transl Med. 2016 Dec 20;14:341. doi: 10.1186/s12967-016-1097-0 (PMC5168869; doi:10.1186/s12967-016-1097-0)
Supplement: Supplementary file 6 — Additional file 6: Figure S1. Derivation of iPS cells in defined culture conditions. [file 12967_2016_1097_MOESM6_ESM.pdf]

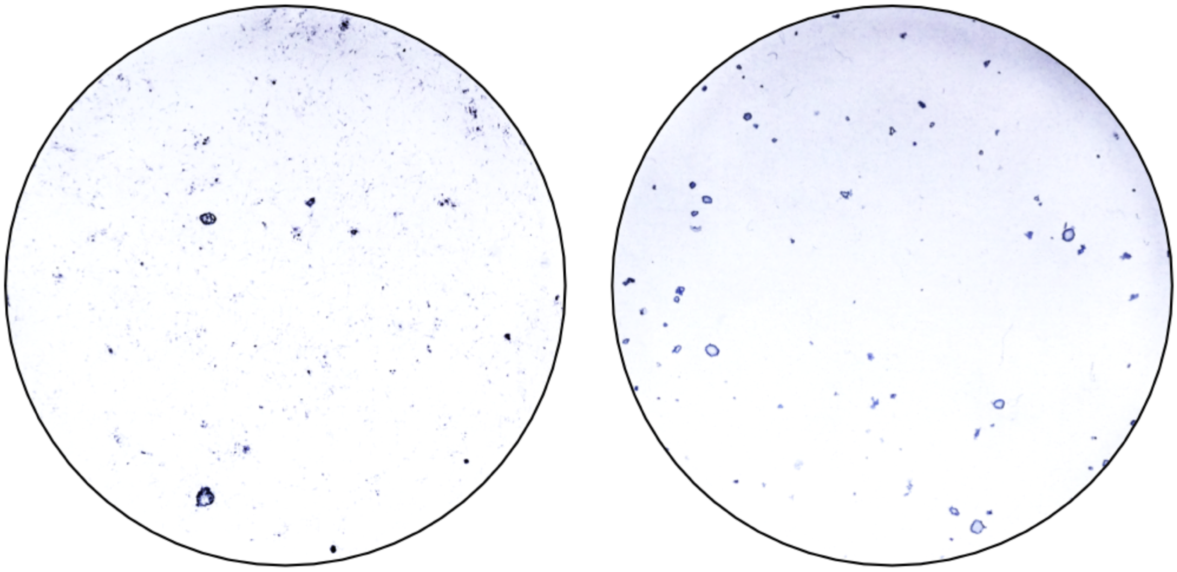

Figure S1. Derivation of iPS cells in defined culture conditions

Alkaline phosphatase stainings of iPS cells generated from HFF-1 fibroblasts and urinary epithelial cells. Somatic cells were seeded on cell culture vessels coated with Laminin-511 (fibroblasts) or Collagen I (for epithelial cells), and transfected with reprogramming episomal vectors with use of L64-PEI reagent.
