# Supplementary material for: Directed differentiation of human iPSC into insulin producing cells is improved by induced expression of PDX1 and NKX6.1 factors in IPC progenitors
Source: J Transl Med. 2016 Dec 20;14:341. doi: 10.1186/s12967-016-1097-0 (PMC5168869; doi:10.1186/s12967-016-1097-0)
Supplement: Supplementary file 7 — Additional file 7: Table S6. Composition of media used for derivation of the definitive endoderm from iPS cells. [file 12967_2016_1097_MOESM7_ESM.pdf]

Table S6. Composition of media used for derivation of the Definitive Endoderm from iPS cells.

**DE1**	RPMI-1640, Foetal Bovine Serum 2%
**DE2**	RPMI-1640, Human Serum 2%
**DE3**	RPMI-1640, human recombinant Albumin 1 mg/mL, human recombinant Insulin 10 μg/mL, human recombinant Transferrin 10 μg/mL, sodium selenite 20nM
**DE4**	RPMI-1640, human recombinant Albumin 1 mg/mL, human recombinant Insulin 10 μg/mL, human recombinant Transferrin 10 μg/mL, chemically defined lipid concentrate 1:50 dilution, ascorbic acid 12.5 μg/mL
