# Supplementary material for: Directed differentiation of human iPSC into insulin producing cells is improved by induced expression of PDX1 and NKX6.1 factors in IPC progenitors
Source: J Transl Med. 2016 Dec 20;14:341. doi: 10.1186/s12967-016-1097-0 (PMC5168869; doi:10.1186/s12967-016-1097-0)
Supplement: Supplementary file 8 — Additional file 8: Table S7. Comparison of relative SOX17, FOXA2 and CXCR4 expression levels in definitive endoderm cells by means of quantitative RT-PCR. [file 12967_2016_1097_MOESM8_ESM.pdf]

Table S7. Comparison of relative SOX17, FOXA2 and CXCR4 expression levels in Definitive Endoderm cells by means of quantitative RT-PCR

**Sample**	Mean C_T values				PCR Efficiencies				Target gene expression relative to iPS cells				Mean Relative expression		
	SOX17	FOXA2	CXCR4	18S rRNA	SOX17	FOXA2	CXCR4	18S rRNA	SOX17	FOXA2	CXCR4		SOX17	FOXA2	CXCR4
**Undifferentiated iPS cells**	34,590	32,081	30,437	15,336	1,781	1,763	1,795	1,717	0,515	1,034	0,481				
	34,473	31,181	30,669	15,277	1,761	1,770	1,793	1,733	0,534	1,701	0,403				
	32,410	32,464	27,803	15,273	1,863	1,811	1,861	1,809	1,849	0,793	2,340				
	32,154	32,479	27,748	15,118	1,846	1,821	1,867	1,824	1,968	0,717	2,207				
**iPS cells differentiated**	32,681	35,624	33,942	22,701	1,823	1,798	1,844	1,811	131,590	10,203	4,493	**Average**	**160,35**	**37,00**	**473,54**
**in DE1 medium**	33,297	37,059	33,580	22,614	1,788	1,813	1,854	1,825	86,155	4,140	5,331	**SD**	143,28	31,83	719,27
	35,359	33,398	32,851	23,466	1,843	1,854	1,863	1,819	41,204	60,301	13,869	**SEM**	58,50	13,00	293,64
	36,088	36,427	32,996	23,563	1,839	1,857	1,840	1,811	28,113	10,603	13,441				
	31,356	32,637	24,667	22,779	1,854	1,817	1,872	1,815	307,027	62,835	1413,940				
	31,101	32,410	24,739	22,825	1,844	1,815	1,857	1,835	368,011	73,897	1390,174				
**iPS cells differentiated**	30,929	36,277	33,832	16,905	1,849	1,804	1,837	1,895	11,972	0,219	0,152	**Average**	**120,81**	**35,42**	**27,26**
**in DE2 medium**	32,633	34,254	33,787	16,960	1,855	1,792	1,856	1,828	4,419	0,750	0,161	**SD**	132,25	40,42	31,33
	24,353	24,700	22,477	15,000	1,841	1,803	1,851	1,829	204,187	67,321	52,651	**SEM**	66,12	20,21	15,67
	24,060	24,681	22,496	15,126	1,836	1,826	1,843	1,827	262,662	73,387	56,095				
**iPS cells differentiated**	29,741	35,888	33,158	21,066	1,823	1,792	1,864	1,789	293,192	3,292	2,745	**Average**	**121,20**	**21,02**	**89,75**
**in DE3 medium**	30,550	34,996	33,093	21,001	1,836	1,751	1,857	1,809	172,972	5,375	2,748	**SD**	95,81	25,63	130,33
	33,596	32,031	32,282	21,877	1,806	1,808	1,830	1,817	46,349	52,588	7,635	**SEM**	39,11	10,46	53,21
	33,172	31,798	31,775	21,737	1,816	1,790	1,859	1,821	55,080	55,546	9,591				
	28,437	31,383	22,103	17,320	1,849	1,810	1,834	1,819	69,067	5,100	264,183				
	27,921	31,641	22,116	17,251	1,870	1,797	1,874	1,819	90,533	4,203	251,603				
**iPS cells differentiated**	31,640	35,329	33,393	22,614	1,826	1,794	1,859	1,816	234,407	11,548	5,982	**Average**	**167,12**	**32,24**	**615,64**
**in DE4 medium**	34,034	36,836	34,756	22,844	1,853	1,812	1,847	1,810	63,300	5,416	2,965	**SD**	140,61	20,64	943,03
	35,836	34,847	33,055	23,719	1,827	1,854	1,838	1,816	35,917	29,690	14,221	**SEM**	57,41	8,42	384,99
	35,324	34,098	33,754	23,602	1,847	1,872	1,848	1,817	45,633	43,173	8,632				
	31,289	32,725	23,873	22,312	1,851	1,815	1,858	1,804	241,926	45,152	1744,474				
	30,653	32,409	23,835	22,431	1,860	1,814	1,868	1,793	381,562	58,482	1917,575				
